# Designing and Calculating the Nonlinear Elastic Characteristic of Longitudinal–Transverse Transducers of an Ultrasonic Medical Instrument Based on the Method of Successive Loadings

**DOI:** 10.3390/ma15114002

**Published:** 2022-06-04

**Authors:** Huu-Dien Nguyen, Shyh-Chour Huang

**Affiliations:** 1 Department of Mechanical Engineering, National Kaohsiung University of Science and Technology, No. 415, Jiangong Rd., Sanmin Dist, Kaohsiung City 807618, Taiwan; nh.dien@hutech.edu.vn; 2 Institute of Engineering, HUTECH University, No. 475A, Dien Bien Phu Rd., Binh Thanh Dist, Ho Chi Minh City 700000, Vietnam

**Keywords:** numerical method, method of successive loadings, nonlinear problem, linearized system of equations, plain curved rod, longitudinal–transverse transducer, nonlinear elastic characteristic

## Abstract

This paper presents a numerical method for studying the stress–strain state and obtaining the nonlinear elastic characteristics of longitudinal–transverse transducers. The authors propose a mathematical model that uses a direct numerical solution of the boundary value problem based on the plain curved rod equations in Matlab. The system’s stress–strain state and nonlinear elastic characteristic are obtained using the method of successive loadings based on the curved rod’s linearized equations. For most ultrasonic instruments, the operating frequency of ultrasonic vibrations is close to 20 kHz. On the other hand, the received own oscillation frequencies are close to the working range. Using the method of successive loadings in the mathematical complex Matlab, a numerical calculation of the stress–strain state of a flat, curved rod at large displacements has been carried out. The proposed model can be considered an initial approximation to the solution of the spatial problem of the longitudinal–torsional transducer.

## 1. Introduction

**Figure 1:**
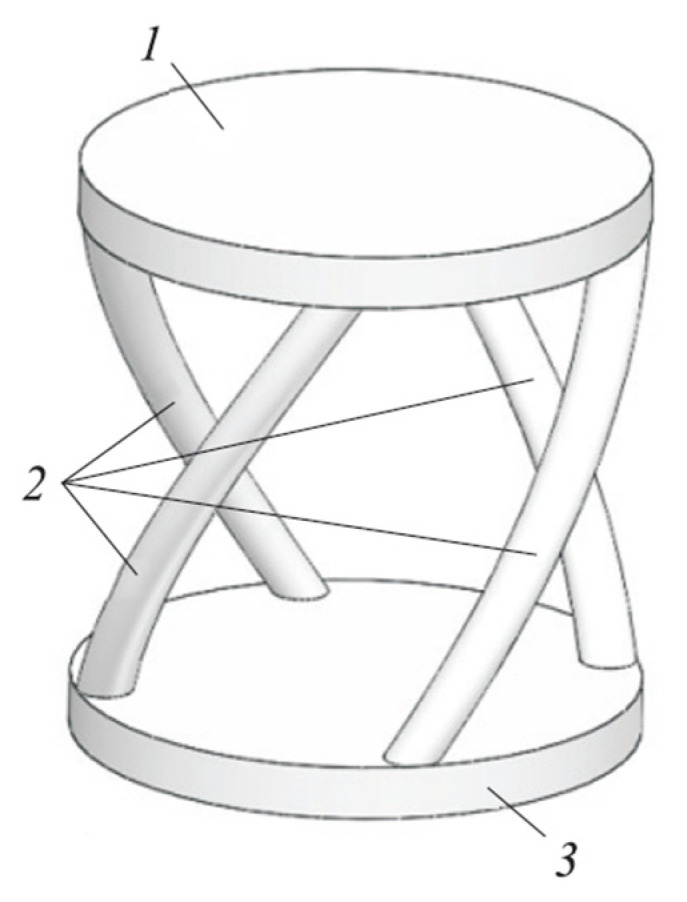
Structure and principle of operation of the longitudinal–transverse transducer: (1) linear displacement entry platform; (2) screw rods; (3) exit platform, performing rotary displacement.

Since its inception, ultrasound-based diagnostic techniques have been increasingly widely used in medicine. Equipping ultrasound machines is the most basic investment in modern medical examination and treatment facilities. The larger the facility, the more advanced and expensive are its equipment. In addition to its diagnostic significance, ultrasound has low risks, wide application, and it is also the main source of income for conventional medical facilities. In the ultrasound machine, the transducer and its model (see Figure 1) are the parts that function the most, receiving and processing the initial signal. The probe set in this research is applied in the medical field. It mediates the analysis and transmission of data from the patient’s body contact and then outputs the data and encodes the image on the computer screen. Of course, in this research, only its mechanical behavior was studied [1,2,3,4,5].

This paper considers a geometrically nonlinear theory of rods. In this theory, the rod is modeled by a one-dimensional curve that has distributed inertial and stiffness characteristics. The deformations of bending, shear, and tension–compression are taken into account, and no restrictions are imposed on the values of displacements and turns. In the plane problem, each point of such a rod has three degrees of freedom—two translational and one rotational. This theory is called the Timoshenko Theory, often known as Geometrically Exact Theory [6,7,8,9,10].

Considering all rod stiffnesses (tension–compression, shear, and bending) is necessary when calculating highly loaded elements of structures, such as high-rise, multi-story buildings, offshore oil platforms, chimneys, and masts. The linear theory of perfectly elastic rods is usually used as a design model. However, geometric nonlinearity must be taken into account, considering that heavy loads lead to large changes in the rod’s geometry [11,12,13].

The general nonlinear formulation of the problems of the statics and stability of rods and their systems has not yet been widely implemented in software systems used in design practice. Further, the accounting for geometric nonlinearity in these complexes is based on a simplified formulation that only considers the effect of the compressive force on the bending stiffness of the rod (i.e., the so-called calculation according to the deformed scheme). Thus, constructing more complex nonlinear models will make it possible to obtain a more accurate solution in some cases [14].

Presently, the calculation of stability is carried out using approximate methods, which do not accurately assess the real stresses in structures. In addition, all computer programs used to calculate the stability of structures ignore the tensile-compressive stiffness and only take into account either the bending stiffness according to the Euler formula or the bending and shear stiffness according to the Engesser formula [15].

Understanding longitudinal vibrations of rods is a classical structural mechanics theme. The standard problem involving a rod with uniform cross-section excited by continuous or point-loads is described in several textbooks, such as in Clough and Penzien, and Rao. In these references the governing equation is shown to be a linear partial differential equation that is solved, for instance, by the method of separation of variables, or by the boundary element method. A relevant development of this problem relates to vibrations of rods with variable cross-sections. In this context, Eisenberger proposed a method for obtaining the exact longitudinal vibration frequencies of tapered rods. Specifically, he considered the case of rods with polynomial variations of the cross-sectional area. Other exact solutions were derived by Abrate who studied the vibration of conical rods, and by Bapat who considered exponential and catenoidal rods. Further, Kumar investigated the case of rods having polynomial, as well as sinusoidal variations of the cross-sectional area by representing the mode shapes in terms of Bessel, Neumann, and trigonometric functions. Nonlinear problems were considered by Wie and Gui-tong and Cveticanin and Uzelac. Specifically, Wei and Gui-tong applied an inverse scattering method for analyzing strain solitons in a rod with nonlinear elastic constitutive law [16,17,18,19].

Furthermore, considerable research is devoted to the field of rod stability and nonlinear deformation. Researchers were engaged in solving structural mechanics problems in a variational setting. Noteworthily, Eliseev VV also made a significant contribution to the development of the nonlinear theory of rods [20].

## 2. The Method of Successive Loadings (MSL)

When solving problems in loading structures comprising rod elements, one often has to deal with large displacements of these elements (i.e., commensurate with the length of the rod). Several methods for solving nonlinear problems applicable to the calculation of plates, shells, membranes, and rods have been developed in the mechanics of deformable solids. This section describes a relatively simple MSL to solve the plane problems of rods’ mechanics, which is accurate enough for everyday engineering practice. The results of numerical studies on rods exposed to different loads are presented, of which exact analytical solutions are available in the literature. In addition, the accuracy of the proposed method of solving such problems is demonstrated. The described method for numerically investigating flat rods’ deep deformation can be easily applied to studying constructions of spatial rods of arbitrarily complex geometry. This section may be of interest to specialists in the field of rod mechanics [21,22,23,24,25].

This variant of numerical solution for nonlinear equations leads to linear equations for each discrete increase in load. For the *m*th loading of the rod, the external force *F*^(*m*)^ = *kF*, where m is the loading step number; *k* is a parameter that determines the part of the total load at each loading step, *k* = 1/m (m-the number of loading steps). Thus, the solution to the deformation of the rod at large displacements is replaced by a consistent solution of a number of problems on the deformation of the rod at small displacements (i.e., linear problems).

Before considering a flat rod, allow us to show how the system of MSL equations can be obtained using the example of a spatial rod. We will take, as a basis for further research, the nonlinear system of equilibrium equations spatially curved rod in connected axes [26,27,28,29,30]:

(1) $$\begin{array}{l} \frac{dQ}{d\eta }+\chi \times Q+q+\sum_{i=1}^{n}F^{\left(1\right)}\delta (\eta -\eta_{i})=0;\\ \frac{dM}{d\eta }+\chi \times M+e_{1}\times Q+\mu +\sum_{\nu =1}^{n}T^{\left(\nu \right)}\delta (\eta -\eta_{\nu })=0;\\ L_{1}\frac{d\theta }{d\eta }+L_{2}\chi_{0}^{\left(1\right)}-A^{-1}M=0;\\ \frac{du}{d\eta }+\chi \times u+\left(l_{11}-1\right)e_{1}+l_{21}e_{2}+l_{31}e_{3}=0;\\ M=A(\chi -\chi_{0}^{\left(1\right)}), \end{array}$$

where *Q*, *M*, *u* are vectors of internal forces, moments, and displacements of points of the centerline of the rod, respectively; η is a dimensionless arc coordinate, η=s/l (*s* is the dimensional coordinate; *l* is the rod length); χ and $\chi_{0}^{\left(1\right)}$ are vectors, whose components are the curvature of the rod’s centerline after loading living and in a natural state; *q* and μ are distributed forces and moments; $F^{\left(i\right)}$ and $T^{\left(\nu \right)}$ are concentrated forces and moments; $\delta \left(\eta -\eta_{i}\right)$, $\delta \left(\eta -\eta_{\nu }\right)$ are generalized Dirac functions defined in the corresponding coordinates, $\eta_{i}$, $\eta_{\nu }$; θ is the vector of the angles of rotation of the connected axes relative to the position in the natural (unloaded) state; $\theta =\vartheta_{i}e_{i}$ ($\vartheta_{i}$ refers to the components of the vector of rotation angles; $e_{i}$ is the unit vectors of the bound deformed main basis); *A* refers to the diagonal matrix dimensionless torsional stiffnesses; $l_{ij}$ are the elements of the transformation matrix of basis vectors, *L.* The matrices $L_{1}$ and $L_{2}$ are included in the system of Equation (1), as well as the matrix *L*.

We obtain the system of MSL equations to measure the first equation for internal forces.

At the first step of loading:

(2) $$F=\Delta F^{\left(1\right)}$$

here and below, the superscript corresponds to the loading step number. We will take all external forces,

(3) $$F=q+\sum_{i=1}^{n}F^{\left(i\right)}\delta (\eta -\eta_{i})$$

and all external moments,

(4) $$T=q+\sum_{\nu =1}^{k}T^{\left(\nu \right)}\delta (\eta -\eta_{i}),$$

Then,

(5) $$\frac{d\Delta Q^{\left(1\right)}}{d\eta }+\left(\chi_{0}+\Delta \chi^{\left(1\right)}\right)\Delta Q^{\left(1\right)}+\Delta F^{\left(1\right)}=0$$

where $\Delta Q^{\left(1\right)}$ is the increment of the internal force by the first step of loading; $\chi_{0}$ is the initial curvature; and $\Delta \chi^{\left(1\right)}$ is the change in curvature.

For linearized Equation (5), it is necessary to take $\Delta \chi^{\left(1\right)}\approx 0$ (i.e., assume that the change in curvature at the first step is small compared to the initial curvature $\chi_{0}$). Then, the linearized equation for the forces at the first step of loading will have the following form:

(6) $$\frac{d\Delta Q^{\left(1\right)}}{d\eta }+\chi_{0}\times \Delta Q^{\left(1\right)}+\Delta F^{\left(1\right)}=0.$$

At the second loading step, we write the equation for the forces in absolute values:

(7) $$\frac{d\left(\Delta Q^{\left(1\right)}+\Delta Q^{\left(2\right)}\right)}{d\eta }+\left(\chi_{0}+\Delta \chi^{\left(1\right)}+\Delta \chi^{\left(2\right)}\right)\times \left(\Delta Q^{\left(1\right)}+\Delta Q^{\left(2\right)}\right)+\Delta F^{\left(1\right)}+\Delta F^{\left(2\right)}=0.$$

By eliminating from this equation the terms which constitute identity (6), neglecting infinitesimals, and linearizing this equation (setting $\Delta \chi^{\left(2\right)}\times \Delta Q^{\left(2\right)}\approx 0$), we get the following equation:

(8) $$\frac{d\left(\Delta Q^{\left(2\right)}\right)}{d\eta }+\left(\chi_{0}+\Delta \chi^{\left(1\right)}\right)\times \Delta Q^{\left(2\right)}+\Delta \chi^{\left(2\right)}\times \Delta Q^{\left(1\right)}+\Delta F^{\left(2\right)}=0.$$

At the *m*th load step (assuming the number step), we will have a vector equation for internal forces:

(9) $$\frac{d\left(\Delta Q^{\left(m\right)}\right)}{d\eta }+\left(\chi_{0}+\sum_{i=1}^{m-1}\Delta \chi^{\left(i\right)}\right)\Delta Q^{\left(m\right)}+\Delta \chi^{\left(m\right)}\sum_{i=1}^{m-1}\Delta Q^{\left(1\right)}+\Delta F^{\left(m\right)}=0.$$

The remaining equations of system (1) are reduced to a similar linearized form. Thus, it is possible to obtain a system of ordinary linearized differential equations of MSL, which describes the behavior of a spatially curvilinear rod at the *m*th loading step. In particular, we present it by replacing vector products with vector matrices:

(10) $$\begin{array}{l} \frac{d\Delta Q^{\left(m\right)}}{d\eta }+A_{\chi }^{\left(m-1\right)}\Delta Q^{\left(m\right)}+A_{Q}^{\left(m-1\right)}\Delta \chi^{\left(m\right)}=-\Delta F;\\ \frac{d\Delta M^{\left(m\right)}}{d\eta }+A_{\chi }^{\left(m-1\right)}\Delta M^{\left(m\right)}+A_{M}^{\left(m-1\right)}\Delta \chi^{\left(m\right)}+A_{1}\Delta Q^{\left(m\right)}=-\Delta T;\\ \frac{d\Delta \theta^{\left(m\right)}}{d_{\eta }}+A_{\chi }^{\left(m-1\right)}\Delta \theta^{\left(m\right)}-\Delta \chi^{\left(m\right)}=0;\\ \frac{d\Delta u^{\left(m\right)}}{d\eta }+A_{\chi }^{\left(m-1\right)}\Delta u^{\left(m\right)}+A_{1}\Delta \theta^{\left(m\right)}=0;\\ M^{\left(m\right)}=A\Delta \chi^{\left(m\right)}, \end{array}$$

where $Q^{\left(m\right)}$, $\Delta M^{\left(m\right)}$, $\Delta \theta^{\left(m\right)}$, and $\Delta u^{\left(m\right)}$ are the increment vectors of the internal force, internal moment, angular displacements, and linear displacements at the *m*th loading step, respectively.

In the system of Equation (10):

(11) $$\begin{array}{l} A_{\chi }^{\left(m-1\right)}=\left[\begin{array}{ccc} 0 & -\chi_{3}^{\left(m-1\right)} & \chi_{2}^{\left(m-1\right)}\\ \chi_{3}^{\left(m-1\right)} & 0 & -\chi_{1}^{\left(m-1\right)}\\ -\chi_{2}^{\left(m-1\right)} & \chi_{1}^{\left(m-1\right)} & 0 \end{array}\right];\\ A_{Q}^{\left(m-1\right)}=\left[\begin{array}{ccc} 0 & Q_{3}^{\left(m-1\right)} & -Q_{2}^{\left(m-1\right)}\\ -Q_{3}^{\left(m-1\right)} & 0 & Q_{1}^{\left(m-1\right)}\\ -Q_{2}^{\left(m-1\right)} & -Q_{1}^{\left(m-1\right)} & 0 \end{array}\right];\\ A_{M}^{\left(m-1\right)}=\left[\begin{array}{ccc} 0 & M_{3}^{\left(m-1\right)} & -M_{2}^{\left(m-1\right)}\\ -M_{3}^{\left(m-1\right)} & 0 & M_{1}^{\left(m-1\right)}\\ M_{2}^{\left(m-1\right)} & -M_{1}^{\left(m-1\right)} & 0 \end{array}\right];\\ A_{1}=\left[\begin{array}{ccc} 0 & 0 & 0\\ 0 & 0 & -1\\ 0 & 1 & 0 \end{array}\right], \end{array}$$

where

$$\chi_{j}^{\left(m-1\right)}=\chi_{j0}+\sum_{k=1}^{m-1}\Delta \chi_{j}^{\left(k\right)};Q_{j}^{\left(m-1\right)}=\Delta Q_{j}^{k};M_{j}^{\left(m-1\right)}=\sum_{k=1}^{m-1}\Delta M_{j}^{\left(k\right)}.$$

Based on System (10) for spatially curved rods, you can get the system of MSL equations for a flat rod.

Its behavior is not described by twelve state vector components but rather by six equations:

(12) $$\begin{array}{l} \frac{d\Delta Q_{1}^{\left(m\right)}}{d\eta }-\chi_{3}^{\left(m-1\right)}\Delta Q_{2}^{\left(m\right)}-Q_{2}^{\left(m-1\right)}\Delta M_{3}^{\left(m\right)}=-\Delta F_{1};\\ \frac{d\Delta Q_{2}^{\left(m\right)}}{d\eta }+\chi_{3}^{\left(m-1\right)}\Delta Q_{1}^{\left(m\right)}+Q_{1}^{\left(m-1\right)}\Delta M_{3}^{\left(m\right)}=-\Delta F_{2};\\ \frac{d\Delta M_{3}^{\left(m\right)}}{d\eta }-\Delta Q_{2}^{\left(m\right)}=-\Delta T_{3};\\ \frac{d\Delta \vartheta_{3}^{\left(m\right)}}{d\eta }-\frac{1}{A_{33}}\Delta M_{3}^{\left(m\right)}=0;\\ \frac{d\Delta u_{1}^{\left(m\right)}}{d\eta }-\chi_{3}^{\left(m-1\right)}\Delta u_{2}^{\left(m\right)}=0;\\ \frac{d\Delta u_{2}^{\left(m\right)}}{d\eta }+\chi_{3}^{\left(m-1\right)}\Delta u_{1}^{\left(m\right)}+\Delta \vartheta_{3}^{\left(m\right)}=0, \end{array}$$

where $\chi_{3}^{\left(m-1\right)}$ and $Q_{j}^{\left(m-1\right)}$ are the curvature and internal forces accumulated in the rod at the previous (*m*−1) loading steps:

(13) $$\begin{array}{l} \chi_{3}^{\left(m-1\right)}=\chi_{30}+\sum_{i=1}^{m-1}\Delta \chi_{3}^{\left(i\right)},\\ Q_{j}^{\left(m-1\right)}=\sum_{i=1}^{m-1}\Delta Q_{j}^{\left(i\right)},j=1,2; \end{array}$$

where $\Delta \vartheta_{3}^{\left(m\right)}$ is the rotation angle increment at the *m*th loading step.

It should be noted that this kind of system works only under the action of follower forces when there is no so-called load increment associated with a change in the direction of forces with respect to the vectors of the associated basis. For example, in the case of the action of dead forces, the right side of the first three equations will change.

In order to judge the accuracy of MSL, it must be applied to the numerical solution of the problem on the deformation of flat rods that have an exact analytical solution.

To reduce the boundary value problem into a Cauchy problem (to the problem with initial conditions), we use the method of initial parameters. Considering system (12) and representing it in the form of one vector–matrix equation:

(14) $$\frac{dY^{\left(m\right)}(\eta )}{d\eta }+A(\eta )Y^{\left(m\right)}(\eta )=0$$

where $Y^{\left(m\right)}\left(\eta \right)$ is the system state vector, $Y^{\left(m\right)}\left(\eta \right)=\left(\Delta Q_{1}^{\left(m\right)},\;\Delta Q_{2}^{\left(m\right)},\;\Delta M_{3}^{\left(m\right)},\;\Delta \vartheta_{1}^{\left(m\right)},\;\Delta u_{1}^{\left(m\right)},\;\Delta u_{2}^{\left(m\right)}\right);A\left(\eta \right)$ is the system coefficient matrix.

We write the solution to Equation (1) in the following form:

(15) $$Y^{\left(m\right)}(\eta )=K^{\left(m\right)}(\eta )C^{\left(m\right)},$$

where $K^{\left(m\right)}\left(\eta \right)$ is the fundamental decision matrix at the loading step; $C^{\left(m\right)}$ is the vector constant at the *m*th step.

To obtain matrix $K^{\left(m\right)}\left(\eta \right)$, we integrate the homogeneous system of Equation (1) six times with the following initial conditions:

(16) $$Y_{1}^{\left(m\right)}(0)=\left[\begin{array}{c} 1\\ 0\\ 0\\ 0\\ 0\\ 0 \end{array}\right],Y_{2}^{\left(m\right)}(0)=\left[\begin{array}{c} 0\\ 1\\ 0\\ 0\\ 0\\ 0 \end{array}\right],\ldots ,Y_{6}^{\left(m\right)}(0)=\left[\begin{array}{c} 0\\ 0\\ 0\\ 0\\ 0\\ 1 \end{array}\right].$$

Every decision $Y_{i}^{\left(m\right)}\left(\eta \right)$ will be the ith column matrices $K_{i}^{\left(m\right)}\left(\eta \right).$ In accordance with the above boundary conditions at η=0, we have $c_{4}^{\left(m\right)}=c_{5}^{\left(m\right)}=c_{6}^{\left(m\right)}=0.$ For the rest of the three components of the vector $C^{\left(m\right)}$, we obtain three algebraic equations from the conditions for η=1:

(17) $$\{\begin{array}{c} k_{21}^{\left(m\right)}(1)c_{1}^{\left(m\right)}+k_{22}^{\left(m\right)}(1)c_{2}^{\left(m\right)}+k_{23}^{\left(m\right)}(1)c_{3}^{\left(m\right)}=\Delta F;\\ k_{41}^{\left(m\right)}(1)c_{1}^{\left(m\right)}+k_{42}^{\left(m\right)}(1)c_{2}^{\left(m\right)}+k_{43}^{\left(m\right)}(1)c_{3}^{\left(m\right)}=0;\\ k_{51}^{\left(m\right)}(1)c_{1}^{\left(m\right)}+k_{52}^{\left(m\right)}(1)c_{2}^{\left(m\right)}+k_{53}^{\left(m\right)}(1)c_{3}^{\left(m\right)}=\Delta F, \end{array}$$

where $k_{ij}^{\left(m\right)}\left(1\right)$ refers to matrix components $K^{\left(m\right)}\left(1\right)$.

From system (1), we find the remaining three constants, $c_{1}^{\left(m\right)}$,$\;c_{2}^{\left(m\right)},\;c_{3}^{\left(m\right)}$. This way, we can completely form a solution, $Y^{\left(m\right)}\left(\eta \right)$ at the current mth step using Formula (15). This algorithm repeats a predetermined number of steps loading *n*. As a result, we have the stress–strain state of the rod:

(18) $$\begin{array}{l} Q_{i}^{\left(n\right)}(\eta )=\sum_{j=1}^{n}\Delta Q_{i}^{\left(j\right)}(\eta );M_{3}^{\left(n\right)}(\eta )=\sum_{j=1}^{n}\Delta M_{3}^{\left(j\right)}(\eta );\\ \vartheta_{3}^{\left(n\right)}(\eta )=\sum_{j=1}^{n}\Delta \upsilon_{3}^{\left(j\right)}(\eta );u_{xi}^{\left(n\right)}(\eta )=\sum_{j=1}^{n}\Delta u_{xi}^{\left(j\right)}(\eta ),i=1,2. \end{array}$$

**Figure 2:**
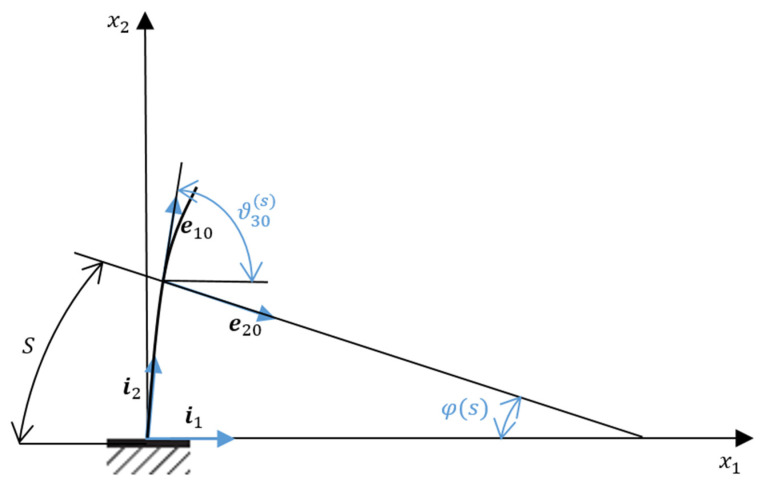
Relationship between the center angle φ(s) and angle $\vartheta_{30}$(s).

Note that internal forces, moments, and rotation angles are simply summed up at each step. Meanwhile, linear displacements must also be summed up on an unchanged Cartesian basis. Therefore, a corresponding transition matrix is formed at each loading step, which occurs in the following manner. First, the transformation matrix $L^{0}$ of the Cartesian basis {*i*} into the associated undeformed $\left\{e_{0}\left(\eta \right)\right\}$ (see Figure 2). Then, the angle between the unit vectors, $i_{1}$ and $i_{10}$, is $\vartheta_{30}\left(\eta \right)=\frac{\pi }{2}-\varphi \left(\eta \right)$, where φ(η)=s/R. Therefore, matrix $L^{0}$ has the following form:

(19) $$L^{0}(\eta )=\begin{array}{c} \begin{array}{c} e_{10}\\ e_{20} \end{array} \end{array}\left[\begin{array}{cc} i_{1} & i_{2}\\ \begin{array}{c} \cos\vartheta_{30}(\eta )\\ \sin\vartheta_{30}(\eta ) \end{array} & \begin{array}{c} \sin\vartheta_{30}(\eta )\\ -\cos\vartheta_{30}(\eta ) \end{array} \end{array}\right]=\begin{array}{c} \begin{array}{c} e_{10}\\ e_{20} \end{array} \end{array}\left[\begin{array}{cc} i_{1} & i_{2}\\ \begin{array}{c} sin\varphi (\eta )\\ \cos\varphi (\eta ) \end{array} & \begin{array}{c} \cos\varphi (\eta )\\ -\sin\varphi (\eta ) \end{array} \end{array}\right].$$

## 3. Applying MSL in the Numerical Method (Matlab) and FEM (Ansys Workbench)

### 3.1. Designing the Model of Longitudinal–Transverse Transducers

This section considers the small vibrations of a spatial helical rod included in the longitudinal–torsional transducer of an ultrasonic medical instrument. An algorithm for determining the natural frequencies and waveforms of system vibrations by the method of initial parameters is developed. Based on this algorithm, the real elastic element of the longitudinal–transverse transducer is calculated using the mathematical package Matlab. The obtained natural frequencies ensure the operation of the ultrasound medical instrument in the resonant mode [31,32,33,34,35].

**Figure 3:**
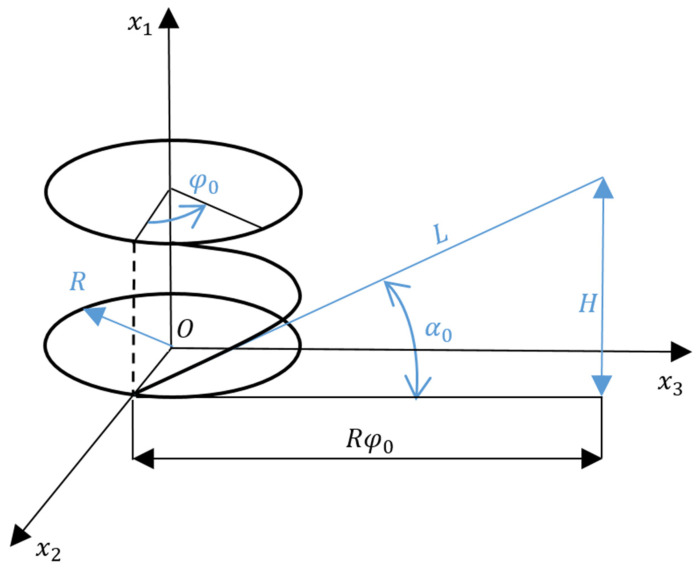
Scheme for calculating the helix.

The helix angle $\alpha_{0}$ is a constant value, so the sweep of a helix on a plane will be represented in the form of a straight line (see Figure 3). From here, we can get formulas relating to the angle lift of the helix ($\alpha_{0})$, the length of the rod (*L*), the height of the rod (*H*), the radius of the circle (*R*), and the angle of twist ($\varphi_{0}$):

(20) $$\begin{array}{l} \alpha_{0}=arctg\frac{H}{R\varphi_{0}};\\ L=\frac{H}{\sin\alpha_{0}}. \end{array}$$

The small free vibrations of a spatial rod in a dimensionless form have the following equation [36,37,38,39,40]:

(21) $$\begin{array}{l} n_{1}\frac{\partial^{2}u}{\partial \tau^{2}}-\frac{\partial \Delta Q}{\partial \varepsilon }-A_{Q}A^{-1}\Delta M-A_{Κ}\Delta Q=0;\\ J\frac{\partial^{2}\vartheta }{\partial \tau^{2}}-\frac{\partial \Delta M}{\partial \varepsilon }-A_{M}A^{-1}\Delta M-A_{Κ}\Delta M-A_{1}\Delta Q=0;\\ \frac{\partial \vartheta }{\partial \varepsilon }+A_{Κ}\vartheta -A^{-1}\Delta M=0;\\ \frac{\partial u}{\partial \varepsilon }+A_{Κ}u+A_{1}\vartheta =0;\\ \Delta M=A\Delta K; \end{array}$$

where

$$\varepsilon =\frac{s}{l};\tau =p_{0}t;p_{0}=\sqrt{\frac{A_{33}}{m_{0}l^{4}}};{\tilde{A}}_{ii}=\frac{A_{ii}}{A_{33}};\tilde{M}=\frac{Ml}{A_{33}};\tilde{Q}=\frac{Ql^{2}}{A_{33}};{\tilde{J}}_{ij}=\frac{J_{ij}}{F_{0}l}.$$

$n_{1}$ is the dimensionless linear mass of the rod; *u* is the displacement vector in the natural coordinate system; Δ*Q* is the vector of internal forces in section; $A_{Q}$ is the matrix of internal forces in a state of equilibrium; $A^{-1}$ is the inverse stiffness matrix of the rod; Δ*M* is the vector of internal moments in the section; $A_{\kappa }$ is the matrix curvature in a state of equilibrium; J refers to the matrix moments of inertia of the section; ϑ is the vector of rotation angles in the natural coordinate system; $A_{M}$ is the matrix of internal moments in the state of equilibrium; $A_{1}$ is an auxiliary identity matrix; *A* refers to the stiffness matrix of the rod; Δ*K* is the matrix of curvature increments; $A_{33}$ is the torsional rigidity of the rod; $m_{0}$ is the mass per unit length of the rod; *s* is the axial coordinate of the section; $p_{0}$ is the factor of dimensionless time; *t* is time; $A_{ii}$ is the dimensional stiffness of the rod; *i* is the number of the natural coordinate axis; *Q* is the dimensional internal forces in the rod section; $J_{ij}$ refers to the moments of inertia of the section; *j* is the number of the natural coordinate axis; $F_{0}$ is the cross-sectional area.

We are looking for a solution to the system of Equation (21) in the following form:

(22) $$\Delta Q=\Delta Q_{0}\left(\varepsilon \right)e^{i\lambda \tau };\Delta M=\Delta M_{0}\left(\varepsilon \right)e^{i\lambda \tau };\vartheta =\vartheta_{0}\left(\varepsilon \right)e^{i\lambda \tau };u=u_{0}\left(\varepsilon \right)e^{i\lambda \tau },$$

where $\Delta Q_{0}$ is an array of increments of internal forces in the section of the rod; λ is the dimensionless natural frequency of the rod; $\Delta M_{0}$ is an array of increments of internal moments; $\vartheta_{0}$ is the vector of initial rotation angles of the section; $u_{0}$ is the vector of initial displacements of the section.

We obtain a system of ordinary differential equations:

(23) $$\begin{array}{l} \frac{dQ_{0}}{d\varepsilon }+A_{Q}A^{-1}M_{0}+A_{Κ}Q_{0}+\lambda^{2}n_{1}u_{0}=0;\\ \frac{dM_{0}}{d\varepsilon }+\left(A_{M}A^{-1}+A_{Κ}\right)M_{0}+A_{1}Q+J\lambda^{2}\vartheta_{0}=0;\\ \frac{d\vartheta_{0}}{d\varepsilon }+A_{Κ}\vartheta_{0}-A^{-1}M_{0}=0;\\ \frac{du_{0}}{d\varepsilon }+A_{Κ}u_{0}+A_{1}\vartheta_{0}=0. \end{array}$$

The system of Equation (23) can be represented in the form of one vector–matrix equation:

(24) $$\frac{d{Ζ}_{0}}{d\varepsilon }+B\left(\varepsilon ,\lambda \right){Ζ}_{0}=0.$$

here

(25) $$\begin{array}{l} {Ζ}_{0}=\left\{\begin{array}{c} \Delta Q_{0}\\ \Delta M_{0}\\ \vartheta_{0}\\ u_{0} \end{array}\right\};\\ Β\left(\varepsilon ,\lambda \right)=\left[\begin{array}{cccc} A_{Κ} & A_{Q}A^{-1} & 0 & \lambda^{2}n_{1}E\\ A_{1} & A_{Μ}A^{-1} & J\lambda^{2} & 0\\ 0 & -A^{-1} & A_{Κ} & 0\\ 0 & 0 & A_{1} & A_{Κ} \end{array}\right], \end{array}$$

where *E* is the elastic modulus of the rod.

The general solution of Equation (24) has the following form:

(26) $$Z_{0}\left(\varepsilon \right)=K\left(\varepsilon \right)C,$$

where K(ε) is the fundamental decision matrix; K(0) = *E*.

To obtain matrix K(ε), we integrate the homogeneous Equation (24) twelve times with the following initial conditions:

(27) $${Ζ}_{0}^{\left(1\right)}=\left\{\begin{array}{c} 1\\ 0\\ 0\\ \begin{array}{c} \overset{\cdot }{\underset{\cdot }{\cdot }}\\ 0 \end{array} \end{array}\right\},{Ζ}_{0}^{\left(2\right)}=\left\{\begin{array}{c} 0\\ 1\\ 0\\ \begin{array}{c} \overset{\cdot }{\underset{\cdot }{\cdot }}\\ 0 \end{array} \end{array}\right\},\ldots ,{Ζ}_{0}^{\left(12\right)}=\left\{\begin{array}{c} 0\\ 0\\ 0\\ \begin{array}{c} \overset{\cdot }{\underset{\cdot }{\cdot }}\\ 1 \end{array} \end{array}\right\}.$$

Based on the rigid pinching of the rod at the ends, we have:

(28) $$\vartheta_{1}=\vartheta_{2}=\vartheta_{3}=u_{1}=u_{2}=u_{3}=0;$$

where $\vartheta_{1}$, $\vartheta_{2}$, $\vartheta_{3}$, and $u_{1}$, $u_{2}$, $u_{3}$ are the components of the vectors ϑ and *u*, respectively.

This means that the six components of vector *C* will be equal to zero since the six components of Vector $Z_{0}$ are equal to zero:

(29) $$c_{7}=c_{8}=\ldots =c_{12}=0.$$

from here,

(30) $$\sum_{j=1}^{6}k_{ij}\left(1\right)c_{j}=0\left(i=7,8,\ldots ,12\right),$$

where $k_{ij}$(1) refers to the elements of the fundamental matrix at ε = 1; $c_{j}$ is the *j*th component of Vector *C*.

Values $\lambda_{j}$ are the natural frequencies of the rod, in which the determinant of System (30) is equal to zero.

After determining the eigenfrequencies of rod $\lambda_{i}$, we find from Equation (24) eigenfunctions $Z_{0}^{\left(j\right)}$ satisfying the boundary conditions of the problem:

(31) $$\frac{d{Ζ}_{0}^{\left(j\right)}}{d\varepsilon }+Β\left(\varepsilon ,\lambda_{j}\right){Ζ}_{0}^{\left(j\right)}=0.$$

Taking the last five equations from the system of linear Equation (30) for each value of natural frequency $\lambda_{j}$, we find that the values $c_{2}^{\left(j\right)},$ $c_{3}^{\left(j\right)}$, …, $c_{6}^{\left(j\right)}$, depending on $c_{1}^{\left(j\right)}$, are determined up to a constant; parameter $c_{0}^{\left(j\right)}$ can be set as equal to one [41,42,43,44,45].

After integrating Equation (31) with the obtained initial vector $C_{j}$, we obtain the corresponding *j*th eigenmode of oscillation.

**Figure 4:**
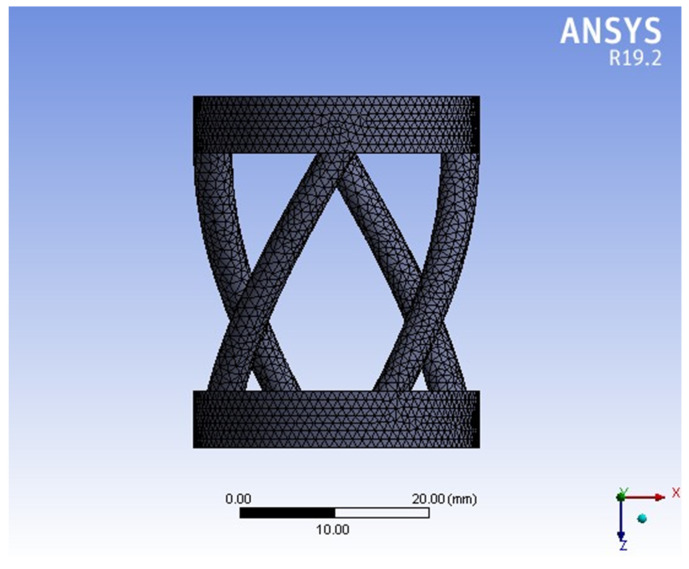
The meshing of the model.

**Figure 5:**
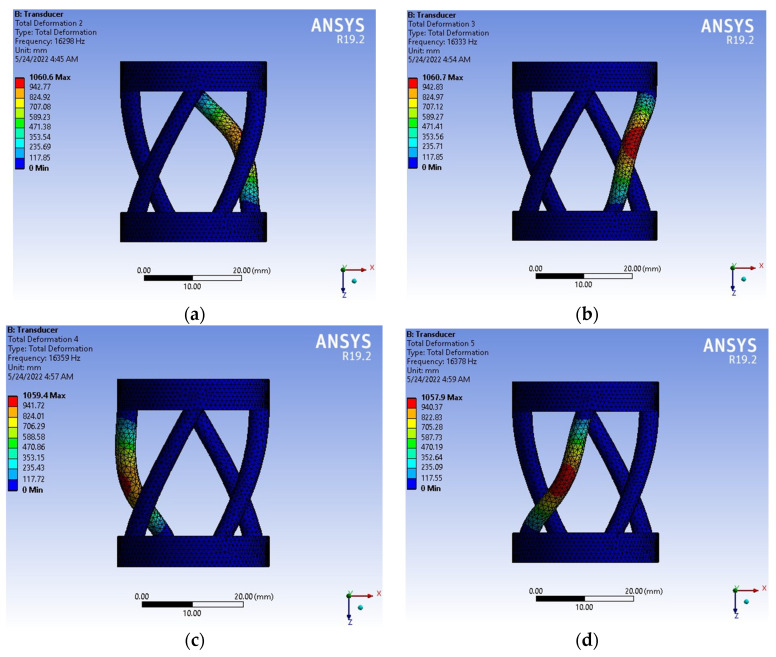

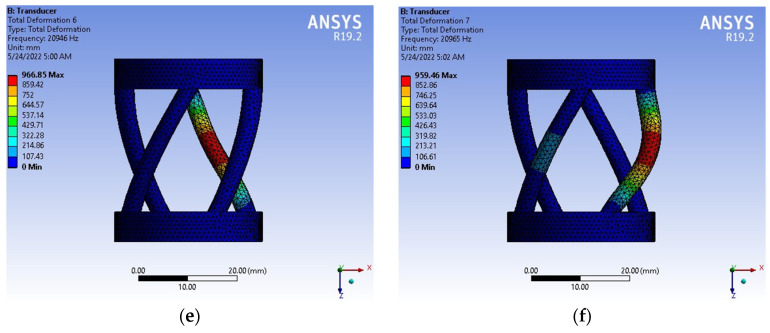
Values of vibration frequency for six modes of model. (**a**) Mode one. (**b**) Mode two. (**c**) Mode three. (**d**) Mode four. (**e**) Mode five. (**f**) Mode six.

The material in this research is polyethylene. Its properties are shown by FEM (Ansys Workbench) in Table 1 and Table 2. Figure 4 shows the meshing of the model, and Figure 5 shows the six values (six modes) of vibration frequency by Ansys Workbench with different rods: $\lambda_{Fem1\left(fre\right)}=16,298\;\mathrm{Hz}$; $\lambda_{Fem2\left(fre\right)}=16,333\;\mathrm{Hz}$; $\lambda_{Fem3\left(fre\right)}=16,359\;\mathrm{Hz}$; $\lambda_{Fem4\left(fre\right)}=16,378\;\mathrm{Hz}$; $\lambda_{Fem5\left(fre\right)}=20,946\;\mathrm{Hz}$; $\lambda_{Fem6\left(fre\right)}=20,965\;\mathrm{Hz}$.

We present the results of the modal analysis of a screw rod with the following initial data: rod’s height *H* = 36 mm; angle of twist $\varphi_{0}=$ π/2; section’s diameter *d* = 4 mm; modulus of elasticity *E* = 2 × 10^5^ MPa (based on Table 2); Poisson’s ratio ν = 0.3 (based on Table 2); dimensionless factor $p_{0}=\sqrt{\frac{A_{3}}{m.L^{4}}}$; material density ρ = 7.85 × 10^6^ kg/mm^3^ (based on Table 1).

In solving the problem in the mathematical package Matlab, the natural frequencies of the rod were obtained: $\lambda_{1}$ = 23.3215; $\lambda_{2}$ = 25.1204; $\lambda_{3}$ = 26.9857. The natural frequencies of rod $\lambda_{i}$ are dimensionless quantities. To convert the dimensionless values of frequencies into hertz, they are multiplied by the dimensionless factor $p_{0}$ and divided by 2 π: $\lambda_{Num.Method1\left(fre\right)}$= 20,835 (Hz); $\lambda_{Num.Method2\left(fre\right)}$ = 20,615 (Hz); $\lambda_{Num.Method3\left(fre\right)}$ = 20,547 (Hz).

For most ultrasonic instruments, the operating frequency of ultrasonic vibrations is close to 20 kHz [46,47,48,49,50]. On the other hand, the obtained natural oscillation frequencies are close to the working range by FEM (Ansys Workbench) and numerical method (Matlab). The most exact value Error_fre_ of FEM is 4.73% at $\lambda_{Fem5\left(fre\right)}$, and the numerical method is 2.74% at $\lambda_{Num.Method2\left(fre\right)}$ (see Table 3) (very good error).

### 3.2. Calculating the Nonlinear Elastic Characteristic of Longitudinal–Transverse Transducers

Let us consider a numerical method that makes it possible to research the stress–strain state of a longitudinal–transverse transducer and obtain its nonlinear elastic characteristic.

The work aims to develop a mathematical model for calculating the stress–strain state of a flat, curved rod at large displacement.

**Figure 6:**
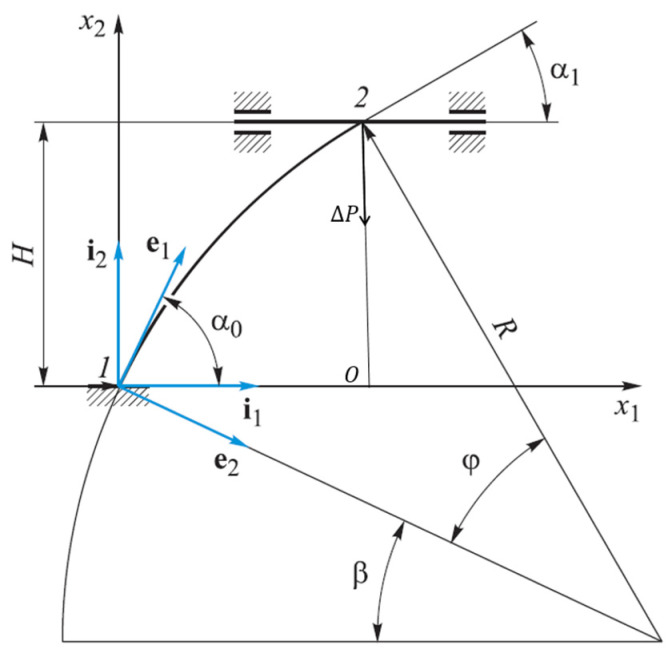
Scheme for determining displacements flat curved rod.

For a flat, curved rod (see Figure 6), we determine the dependence of the vertical displacement (directed along Axis $x_{2}$), where $u_{x2}$ is the upper end of rod 2 on the horizontal displacement (co-directional with Axis $x_{1}$), and $u_{x1}$ is the lower end of rod 1.

We assume that the lower end of the rod moves strictly vertically along Axis $x_{2}$, and its upper end moves in the guides strictly horizontally parallel to axis $x_{1}$ [51]. Further, we also assume that the ends are rigidly clamped; that is, they do not rotate relative to the attachment points. Noteworthily, we neglect the mass of the rod.

The following parameters are specified: height (*H*), starting angle (β), and the angle’s rotation of the arc (*φ*).

The angles between local unit vector $i_{1}$, co-directed with the global axis $x_{1}$, and natural unit vector $e_{1}$, directed tangentially to the arc circles, are defined by the following expressions:

At the beginning of the rod:

(32) $$\alpha_{0}=\frac{\pi }{2}-\beta ;$$

At the end of the rod:

(33) $$\alpha_{1}=\frac{\pi }{2}-\left(\beta +\varphi \right).$$

Height:

(34) H=Rsin(β+φ)−Rsinβ,

where *R* is the radius of the circle.

From relation (23), we express the radius of the circle as follows:

(35) $$R=\frac{H}{\sin\left(\beta +\varphi \right)-\sin\beta }.$$

We get a system from six equations:

(36) $$\begin{array}{l} \frac{dQ_{1}}{d\varepsilon }-K_{30}Q_{2}=0;\\ \frac{dQ_{2}}{d\varepsilon }+K_{30}Q_{1}=0;\\ \frac{dM_{3}}{d\varepsilon }+Q_{2}=0;\\ \frac{d\vartheta_{3}}{d\varepsilon }-\frac{1}{A_{33}}M_{3}=0;\\ \frac{du_{1}}{d\varepsilon }-K_{30}u_{2}=0;\\ \frac{du_{2}}{d\varepsilon }+K_{30}u_{1}-\vartheta_{3}=0, \end{array}$$

where $Q_{1}$ and $Q_{2}$ are internal forces directed along axes $x_{1}$ and $x_{2}$; ε is the axial dimensionless coordinate; $K_{30}$ is the initial curvature of the rod axis; $M_{3}$ is the bending moment; $\vartheta_{3}$ is the rotation angle of the cross-section of the rod; $A_{33}$ is the bending stiffness of the rod; $u_{1}$ and $u_{2}$ are the horizontal and vertical displacements of the rod’s cross-section.

Thus,

(37) ΔP=0

where ΔP—external concentrated force small increment.

## 4. Results and Discussions

One of the methods for solving nonlinear problems is the MSL, in which linear equations describe each discrete increase in the load. For each *m*th step, the external force *F^(m)^ = kF*, where m is the number of the loading step; *k* is a parameter that determines a part of the total load at each stage, and *k* = 1/*n* (*n* is the number of loading steps).

The system of equations for the first step of loading are as follows:

(38) $$\begin{array}{l} \frac{dQ_{1}^{\left(1\right)}}{d\varepsilon }-K_{30}Q_{2}^{\left(1\right)}=0;\\ \frac{dQ_{2}^{\left(1\right)}}{d\varepsilon }+K_{30}Q_{1}^{\left(1\right)}=0;\\ \frac{dM_{3}^{\left(1\right)}}{d\varepsilon }+Q_{2}^{\left(1\right)}=0;\\ \frac{d\vartheta_{3}^{\left(1\right)}}{d\varepsilon }-\frac{1}{A_{33}}M_{3}^{\left(1\right)}=0;\\ \frac{du_{1}^{\left(1\right)}}{d\varepsilon }-K_{30}u_{2}^{\left(1\right)}=0;\\ \frac{du_{2}^{\left(1\right)}}{d\varepsilon }+K_{30}u_{1}^{\left(1\right)}-\vartheta_{3}^{\left(1\right)}=0. \end{array}$$

For each subsequent *m*th step of loading, the system of equations are as follows:

(39) $$\begin{array}{l} \frac{dQ_{1}^{\left(m\right)}}{d\varepsilon }-K_{3}^{\left(m-1\right)}Q_{2}^{\left(m\right)}-Q_{2}^{\left(m-1\right)}\Delta K_{3}^{\left(m\right)}=0;\\ \frac{dQ_{2}^{\left(m\right)}}{d\varepsilon }+K_{3}^{\left(m-1\right)}Q_{1}^{\left(m\right)}+Q_{1}^{\left(m-1\right)}\Delta K_{3}^{\left(m\right)}=0;\\ \frac{dM_{3}^{\left(m\right)}}{d\varepsilon }+Q_{2}^{\left(m\right)}=0;\\ \frac{d\vartheta_{3}^{\left(m\right)}}{d\varepsilon }-\frac{1}{A_{33}}M_{3}^{\left(m\right)}=0;\\ \frac{du_{1}^{\left(m\right)}}{d\varepsilon }-K_{3}^{\left(m-1\right)}u_{2}^{\left(m\right)}=0;\\ \frac{du_{2}^{\left(m\right)}}{d\varepsilon }+K_{3}^{\left(m-1\right)}u_{1}^{\left(m\right)}-\vartheta_{3}^{\left(m\right)}=0, \end{array}$$

where

$$\begin{array}{l} K_{3}^{\left(m-1\right)}=K_{30}+\sum_{k=2}^{m-1}\Delta K_{3}^{\left(m\right)}=K_{30}+\sum_{k=2}^{m-1}\frac{M_{3}^{\left(k\right)}}{A_{33}};\\ \Delta K_{3}^{\left(m\right)}=\frac{M_{3}^{\left(m\right)}}{A_{33}};Q_{j}^{\left(m-1\right)}=\sum_{k=2}^{m-1}\Delta Q_{j}^{\left(k\right)}. \end{array}$$

The boundary conditions for the next step of the problem being solved should be considered.

With a dimensionless axial coordinate ε = 0, the lower end of the rod is rigidly clamped. Therefore, the angle of rotation $\vartheta_{3}$ at the beginning of the section is zero [52].

The origin is set to move along axis $x_{1}$. To solve the problem, it must be projected to the natural axis.

The rotation matrix for transitioning from the Cartesian coordinate system to the natural system has the following form:

(40) $$\begin{array}{l} L\left(\varphi \right)=\left[\begin{array}{cc} \cos\varphi & \sin\varphi \\ -\sin\varphi & \cos\varphi \end{array}\right];\\ \left\{\begin{array}{c} e_{1}\\ e_{2} \end{array}\right\}=L\left(\varphi \right)\left\{\begin{array}{c} i_{1}\\ i_{2} \end{array}\right\}. \end{array}$$

The boundary conditions will be as follows:

(41) $$\vartheta_{3}=0;u_{1}=\Delta u_{0}\sin\alpha_{0};u_{2}=\Delta u_{0}\cos\alpha_{0},$$

where $\Delta u_{0}$ is the small increment of displacement by the end of the rod.

With a dimensionless axial coordinate ε = 1, the upper end of the rod is rigidly clamped. Therefore, the angle of rotation $\vartheta_{3}$ at the end of the segment is zero [53,54,55].

The upper end of the rod can only move horizontally in the guides. Thus, the following condition can be written as follows:

(42) $$u_{x2}=0$$

The force directed along Axis $x_{1}$ must also be zero since the upper end of the rod can move freely in the $x_{2}$ direction:

(43) $$Q_{x2}=0$$

These two boundary conditions are written in the Cartesian coordinate system. By projecting them into the natural axis at the end of the rod, we obtain the following algebraic expressions:

For displacements:

(44) $$u_{x2}=u_{1}\sin\alpha_{1}+u_{2}\cos\alpha_{1};$$

For forces:

(45) $$Q_{2}=Q_{1}\sin\alpha_{1}-Q_{2}\cos\alpha_{1}.$$

Then, the boundary conditions will be as follows:

(46) $$\begin{array}{l} \vartheta_{3}=0;u_{1}\sin\alpha_{1}+u_{2}\cos\alpha_{1}=0;\\ Q_{1}\sin\alpha_{1}-Q_{2}\cos\alpha_{1}=0. \end{array}$$

The resulting boundary conditions must be performed at each step of the solution by the MSL in the software complex Matlab since angles $\alpha_{0}$ and $\alpha_{1}$ remain unchanged throughout solving the problem.

We present the results for calculating a flat, curved rod with the following inputs: *H* = 50 mm; β=π/6; φ=π/6; section’s width *b* = 5 mm; section’s height *h* = 1 mm; Young’s module *E* = 2 × 10^5^ Mpa (based on Table 2).

The radius of the curvature calculated curved-linear rod, calculated by Formula (35): *R_Analysis_* = $\frac{50*10^{-3}m}{\sin\left(\frac{\pi }{6}+\frac{\pi }{6}\right)-\sin\left(\frac{\pi }{6}\right)}\;$ = 0.01566 m.

**Figure 7:**
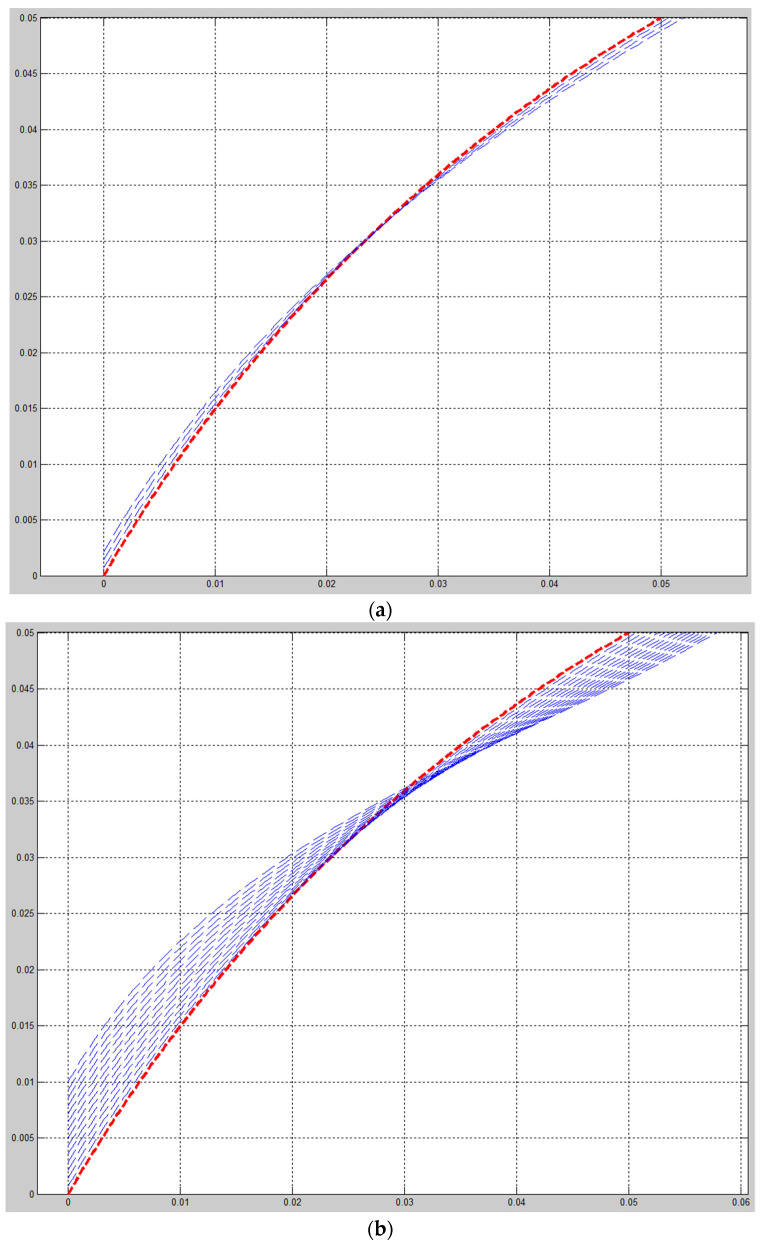

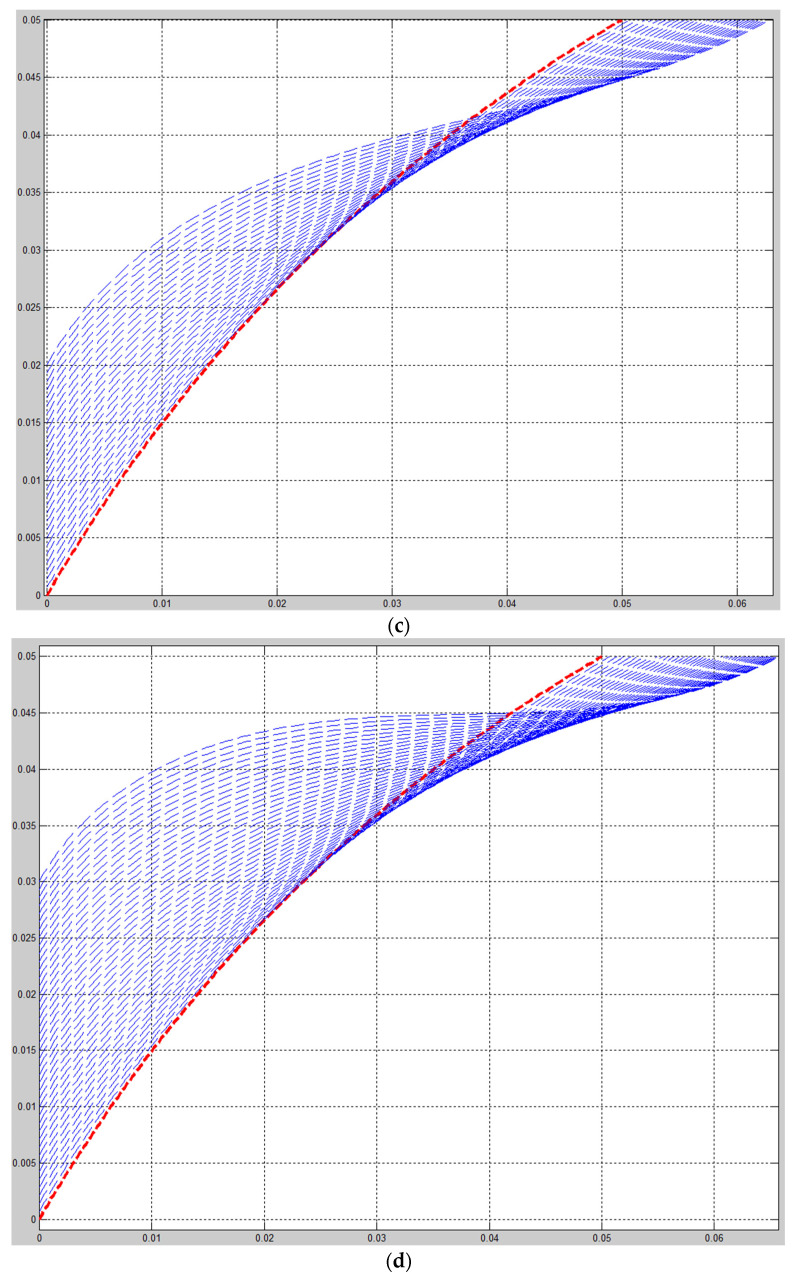

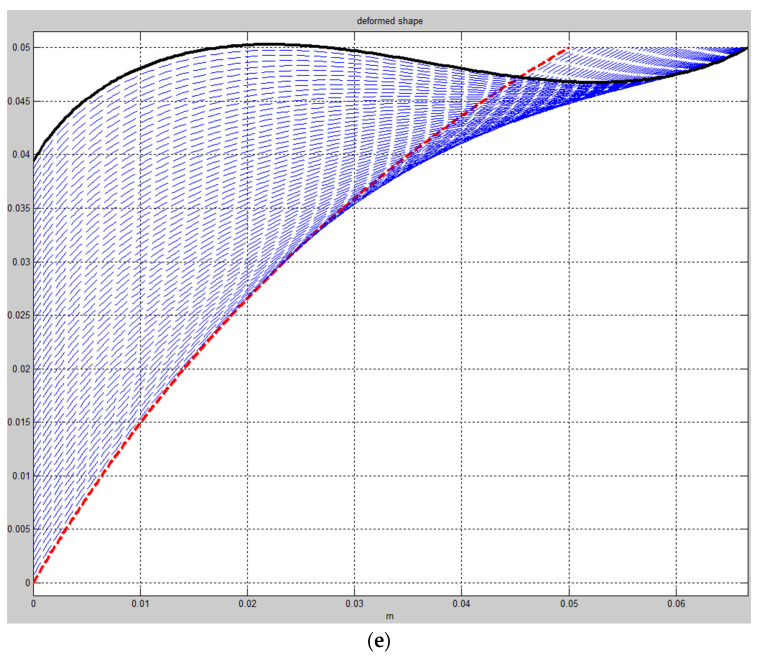
Graphs of the rod deformation axis: (**a**) Beginning process; (**b**) Quarter process; (**c**) Half process; (**d**) Three-fourths process; (**e**) End process.

**Figure 8:**
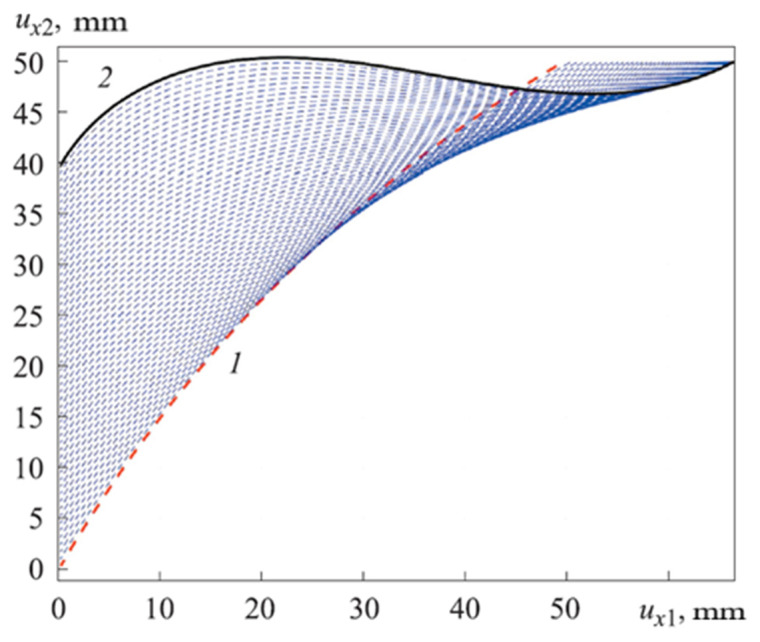
Graphs of the rod deformation axis: 1 is the initial shape of the rod axis; 2 is the shape of the rod axis at the last loading step.

Solving a problem in the mathematical package, Matlab received graphs of the deformation axis of the rod at each step of the loading (see Figure 7 and Figure 8). Figure 7 shows the deformation process of the rod. Figure 8 shows graphs of the rod deformation axis: red curve 1 is the initial shape of the rod axis; black curve 2 is the shape of the rod axis at the last loading step; blue curves are the deformations of the rod. Maximum deformation of rod: u_Num.Method(Max.def)_ = 983.54 mm (see Figure 8); u_Fem1(Max.def)_ = 1060.6 mm, u_Fem2(Max.def)_ = 1060.7 mm, u_Fem3(Max.def)_ = 1059.4 mm, u_Fem4(Max.def)_ = 1057.9 mm, u_Fem5(Max.def)_ = 966.85 mm, u_Fem6(Max.def)_ = 959.46 mm (see Figure 5). The values of deformation of the rod by FEM (Ansys Workbench) and numerical method (Matlab) have an insignificant difference. Maximum (%) error deformation: Error_Max.def2_ =7.27% and minimum (%) error deformation: Error_Max.def5_ =1.73% (see Table 4). That again shows that numerical method is a reliable method in simulation and computational engineering.

**Figure 9:**
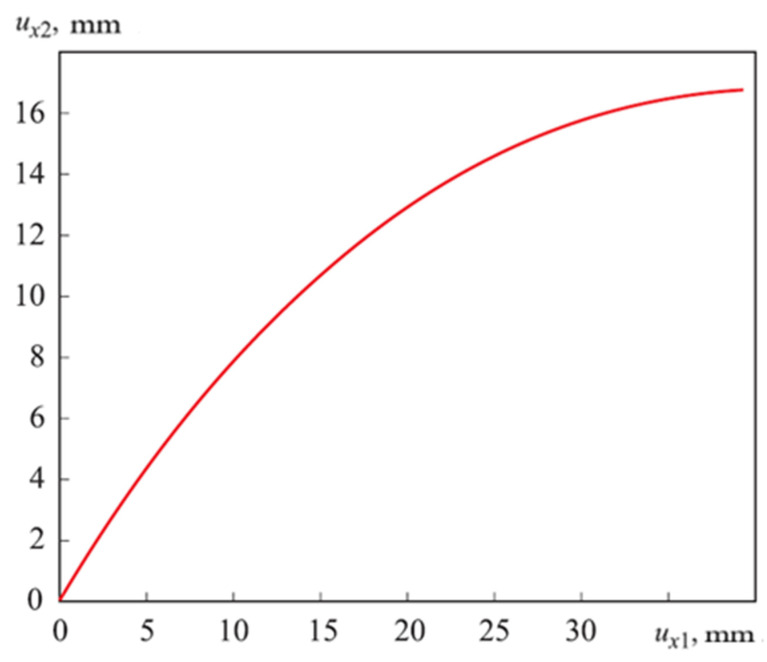
Nonlinear elastic characteristic longitudinal–transverse transducer.

The dependence of the horizontal displacement of the upper end of the rod $u_{x2}$ from the vertical displacement of its lower end of the rod $u_{x1}$ (see Figure 9) is called the nonlinear elastic characteristic of the longitudinal–transverse transducer. The radius of the curvature calculated curved linear rod *R_Num.Method_* = 0.0165 m.

$${\mathrm{Error}}_{\mathrm{Non}.\mathrm{Elas}.\mathrm{Char}}\;=\frac{R_{Num.Method}-R_{Analysis}}{R_{Analysis}}=\frac{0.0165-0.01566}{0.01566}\times 100\%=5.36\%$$

We obtained Error_Non.Elas.Char_ = 5.36%, which proves that the algorithm for developing the MSL in the numerical method (Matlab) is correct.

## 5. Conclusions

The proposed algorithm for solving problems in rod mechanics with large displacements is approximate. However, according to the authors, its accuracy is quite acceptable for engineering practice. This method leverages its simple algorithmization for numerical calculation, in contrast to the solution of similar problems by the “zero” method and the parameter continuation methods. In addition, it should be noted that the geometry and the rod’s material properties can be easily varied, both in terms of the centerline and stiffness variability along the length. The above algorithm for the numerical research on the deep deformation of a flat rod is sufficient and can be easily adapted to solve problems involving static loading of structures in the form of spatially curvilinear rods of arbitrarily complex geometry.

For most ultrasonic instruments, the operating frequency of ultrasonic vibrations is close to 20 kHz. On the other hand, the received own oscillation frequencies are close to the working range. Changing the geometric characteristics of the screw rod and the material’s mechanical properties can achieve such conditions, under which a resonant mode will be implemented.

Using the MSL in the mathematical complex Matlab, a numerical calculation of the stress–strain state of a flat, curved rod at large displacements has been carried out. Based on the results obtained, a nonlinear elastic characteristic of the longitudinal–transverse transducer was constructed.

## Figures and Tables

**Table 1:** Structural steel’s constants by FEM (Ansys Workbench).

Density	7.85 × 10^6^ kg/mm^3^

**Table 2:** Structural steel’s isotropic elasticity by FEM (Ansys Workbench).

Young’s Modulus (MPa)	Poisson’s Ratio	Tensile Yield Strength (MPa)	Tensile Ultimate Strength (MPa)
2 × 10^5^	0.3	250	460

**Table 3:** Value frequencies of vibrations by FEM and the numerical method, and the reality in medicine.

Reality in Medicine (kHz) [48]	20	
$\mathrm{FEM}\;\lambda_{Fem1\left(fre\right)}\;\left(\mathrm{kHz}\right)$	16.298	${\mathrm{Error}}_{\mathrm{fre}}=\frac{20-16.298}{20}\times 100\%=18.51\%$
$\mathrm{FEM}\;\lambda_{Fem2\left(fre\right)}\;\left(\mathrm{kHz}\right)$	16.333	${\mathrm{Error}}_{\mathrm{fre}}=\frac{20-16.333}{20}\times 100\%=18.36\%$
$\mathrm{FEM}\;\lambda_{Fem3\left(fre\right)}\;\left(\mathrm{kHz}\right)$	16.359	${\mathrm{Error}}_{\mathrm{fre}}=\frac{20-16.359}{20}\times 100\%=18.21\%$
$\mathrm{FEM}\;\lambda_{Fem4\left(fre\right)}\;\left(\mathrm{kHz}\right)$	16.378	${\mathrm{Error}}_{\mathrm{fre}}=\frac{20-16.378}{20}\times 100\%=18.11\%$
$\mathrm{FEM}\;\lambda_{Fem5\left(fre\right)}\;\left(\mathrm{kHz}\right)$	20.946	${\mathrm{Error}}_{\mathrm{fre}}=\frac{20.946-20}{20}\times 100\%=4.73\%$
$\mathrm{FEM}\;\lambda_{Fem6\left(fre\right)}\;\left(\mathrm{kHz}\right)$	20.965	${\mathrm{Error}}_{\mathrm{fre}}=\frac{20.965-20}{20}\times 100\%=4.83\%$
$\mathrm{Numerical}\;\mathrm{method}\;\lambda_{Num.Method1\left(fre\right)}$ (kHz)	20.835	${\mathrm{Error}}_{\mathrm{fre}}=\frac{20.835-20}{20}\times 100\%=4.18\%$
$\mathrm{Numerical}\;\mathrm{method}\;\lambda_{Num.Method2\left(fre\right)}$ (kHz)	20.615	${\mathrm{Error}}_{\mathrm{fre}}=\frac{20.615-20}{20}\times 100\%=3.08\%$
$\mathrm{Numerical}\;\mathrm{method}\;\lambda_{Num.Method3\left(fre\right)}$ (kHz)	20.547	${\mathrm{Error}}_{\mathrm{fre}}=\frac{20.547-20}{20}\times 100\%=2.74\%$

**Table 4:** Values of maximum deformation by FEM and numerical method.

FEM (Ansys) (mm)	Numerical Method (Matlab) (mm)	(%) Error_Max.def_
1060.6		${\mathrm{Error}}_{\mathrm{Max}.\mathrm{def}1}\;=\frac{1060.6-983.54}{1060.6}\times 100\%=7.27\%$
1060.7		${\mathrm{Error}}_{\mathrm{Max}.\mathrm{def}2}\;=\frac{1060.7-983.54}{1060.7}\times 100\%=7.27\%$
1059.4	983.54	${\mathrm{Error}}_{\mathrm{Max}.\mathrm{def}3}\;=\frac{1059.4-983.54}{1059.4}\times 100\%=7.16\%$
1057.9		${\mathrm{Error}}_{\mathrm{Max}.\mathrm{def}4}\;=\frac{1057.9-983.54}{1057.9}\times 100\%=7.03\%$
966.85		${\mathrm{Error}}_{\mathrm{Max}.\mathrm{def}5}\;=\frac{966.85-983.54}{966.85}\times 100\%=1.73\%$
959.46		${\mathrm{Error}}_{\mathrm{Max}.\mathrm{def}6}\;=\frac{983.54-959.46}{959.46}\times 100\%=2.50\%$

## Data Availability

Data sharing is not applicable.
